# Data on three-year zooplankton monitoring in ditches of the apple orchard region of Altes Land, Germany

**DOI:** 10.1016/j.dib.2019.103833

**Published:** 2019-03-16

**Authors:** Stefan Lorenz, Axel Mueller

**Affiliations:** Julius Kuehn Institute, Institute for Ecological Chemistry, Plant Analysis and Stored Product Protection, Koenigin-Luise-Strasse 19, 14195 Berlin, Germany

**Keywords:** Agriculture, Biodiversity, Crustacean species, Environmental monitoring, Freshwater monitoring

## Abstract

The data presented in this article are related to the research article ‘Chemical and biological monitoring of the load of plant protection products and of zoocoenoses in ditches of the orchard region Altes Land’ (Süß et al., 2006), which is only available in the German language. The zooplankton data presented here were acquired from four ditches (three ditches were located in apple orchards, and one ditch was located in a grassland region) between 2001 and 2003 (Lorenz & Müller, 2018). This article describes the methods used to determine zooplankton species in the samples. The field data set is publicly available at the OpenAgrar repository under (Lorenz & Müller, 2018). It is related to the field data set of Lorenz et al. (2018) where pesticide monitoring data from the same ditches and time period were presented.

**Table:** Specifications table

Subject area	*Environmental science*
More specific subject area	*Freshwater ecology, ecotoxicology, environmental monitoring*
Type of data	*Table*
How data were acquired	*Grab water samples were filtered over* 150 μm *mesh nets*
Data format	*Excel sheet*
Experimental factors	*Zooplankton species were sampled in agricultural ditches*
Experimental features	*Three sampled ditches were located in an apple orchard, and one ditch was located in a grassland region*
Data source location	*Orchard region of Altes Land, Germany; ditches in Neuenkirchen, Jork and Estebrügge (2 ditches)*
Data accessibility	*The field data set is publicly available at the OpenAgrar repository under*https://doi.org/10.5073/20181213-163305 https://www.openagrar.de/receive/openagrar_mods_00045884
Related research article	[1] A. Süß, G. Bischoff, A. Mueller, L. Buhr, Chemical and biological monitoring of the load of plant protection products and of zoocoenoses in ditches of theorchard region „Altes Land”,Nachrichtenbl. Deut. Pflanzenschutzd., 58 (2006) 28–42.

**Table:** 

**Value of the data** • Long-term monitoring of marine zooplankton is frequently performed, but almost no data from freshwater ditches in agricultural landscapes are available. • The data can be related to previously published data on pesticide contamination generated by either event-driven or weekly integrated sampling. • The data presented here allow for realistic assessment of potential exceedances of environmental quality standards following pesticide application (data linked at the repository). • The data allow other researchers to perform statistical (meta-)analysis on the impact of agriculture on freshwater ecosystems.

## 1 Data

The data presented in this article consist of zooplankton data from monitoring in four ditches of the orchard region of Altes Land, Germany [1]. The data consist of one Excel sheet providing the results of the species determination. The zooplankton field data set consists of 4,710 species observations and is publicly available at the OpenAgrar repository under Ref. [2]. A total of 21 species were recorded (Table 1). Characteristics on ditch temperature, conductivity and dissolved oxygen for each sampling date are presented in Table 2. The data can be related to data on pesticide analysis with description of the pesticide sampling details and study site description [4], which are publicly available at the OpenAgrar repository [3].

**Table 1:** List of zooplankton species recorded in the four ditches from 2001 to 2003. “/” = no sampling.

species	Neuenkirchen			Jork			Estebrügge			Estebrügge grassland		
	2001	2002	2003	2001	2002	2003	2001	2002	2003	2001	2002	2003
*Acroperus harpae*									X	**/**		
*Alona rectangula*	X		X					X	X	**/**	X	X
*Bosmina longirostris*	X			X	X	X	X	X	X	**/**	X	X
Calanoide gen. sp.		X		X	X	X	X	X	X	**/**		X
*Ceriodphnia dubia*							X	X		**/**		
*Ceriodaphnia laticaudata*				X	X	X	X		X	/		X
*Ceriodaphnia quadrangula*				X			X			/		X
*Ceriodaphnia reticulata*				X				X	X	/		X
*Ceriodaphnia rotunda*		X		X	X		X	X		/		
*Ceriodaphnia setosa*				X			X			/		
*Ceriodaphnia* sp.			X		X			X		/	X	
*Chydorus sphaericus*	X	X	X	X	X	X	X	X	X	/	X	X
Cyclopidae gen. sp.	X	X	X	X	X	X	X	X	X	/	X	X
*Daphnia longispina*		X		X	X	X	X	X	X	/	X	X
*Daphnia pulex*		X	X	X	X	X	X	X	X	**/**		
*Diaphanosoma brachyurum*									X	**/**		
*Eurycercus lamellatus*				X	X	X	X		X	**/**		
*Graptoleberis testudinaria*			X				X	X	X	/	X	X
*Harpacticoidae* gen. sp.	X	X	X	X	X	X	X	X	X	/	X	X
*Leptodora kindtii*						X				/		
Ostracoda gen. sp. larvae	X	X	X	X	X	X	X	X	X	/	X	X
Ostracoda gen. sp.	X	X	X	X	X	X	X	X	X	/		
*Peracantha truncata*		X		X	X	X	X	X	X	/	X	X
*Pleuroxus trigonellus*									X	**/**		**X**
*Polyphemus pediculus*				X		X			X	**/**		
*Scapholeberis mucronata*				X		X				/		
*Simocephalus vetulus*		X	X	X	X	X	X	X	X	/	X	X

**Table 2:** Temperature, conductivity and dissolved oxygen values for each sampling date in 2001 and 2003. n.d. = no data are available. Measurements have not been conducted in 2002.

	Temperature [°C]	Conductivity [μS]	Dissolved oxygen [mg/L]
Neuenkirchen			
2001-04-23	11.7	440	14.3
2001-06-05	n.d.	n.d.	n.d.
2001-06-18	19.9	328	12.6
2001-07-16	17.0	427	8.9
2001-08-13	17.4	349	3.2
2001-09-10	16.1	289	3.3
2001-10-07	15.0	346	11.8
2003-04-29	16.4	458	4.0
2003-06-10	18.7	380	5.6
2003-07-22	25.3	437	3.8
2003-09-02	15.1	463	2.2
Jork			
2001-04-23	13.5	764	12.7
2001-06-05	n.d.	n.d.	n.d.
2001-06-18	21.5	417	13.9
2001-07-16	18.9	318	2.8
2001-08-13	16.7	618	2.0
2001-09-10	16.0	461	4.7
2001-10-07	14.7	553	6.1
2003-04-29	14.7	662	9.6
2003-06-10	18.6	667	4.4
2003-07-22	20.5	673	2.4
2003-09-02	13.7	766	1.8
Estebrügge			
2001-04-23	12.7	473	17.8
2001-06-05	n.d.	n.d.	n.d.
2001-06-18	21.5	790	7.6
2001-07-16	17.6	705	2.8
2001-08-13	16.9	890	1.9
2001-09-10	15.9	859	3.1
2001-10-07	13.9	845	3.9
2003-04-29	16.8	827	13.0
2003-06-10	21.3	1300	10.4
2003-07-22	23.5	1426	2.9
2003-09-02	16.5	1330	2.2
Estebrügge grassland			
2003-04-29	16.9	1011	8.1
2003-06-10	18.9	1732	1.9
2003-07-22	26.8	1595	1.6
2003-09-02	17.2	1023	2.5

Zooplankton is an important component of aquatic ecosystems, and zooplankton communities can quickly respond to a wide variety of environmental stressors such as input of nutrients [5], sediment [6] or contaminants [7]. The aforementioned stressors often result from agricultural impacts on freshwater ecosystems which negatively affect zooplankton biodiversity. Hence, zooplankton monitoring in combination with the monitoring of agricultural stressors is a central element to manage and conserve freshwater biodiversity [8].

## 2 Experimental design, materials, and methods

The data were gathered in four representative ditches of the orchard region of Altes Land, Germany, which is close to the Lower Elbe region near the city of Hamburg. Three ditches were located in an apple orchard, and one ditch was located in a grassland region. The site characteristics of the four studied ditches are presented in Lorenz et al. (2018) [4]. Water samples were taken from April to September over three years (2001–2003) to collect zooplankton species. Seven samples were taken at an interval of four weeks in 2001, while four samples at an interval of six weeks were taken in 2002 and 2003. The ditch Estebrügge grassland was not sampled in 2001. Zooplankton was sampled by taking 20 grab samples of 3 L water volume at each ditch (with an interval of 5 m ditch length between the consecutive samples). The grab samples were combined, filtered using a 150 μm mesh net and preserved directly after filtration.

This research did not receive any specific grant from funding agencies in the public, commercial or not-for-profit sectors.

## References

[1] Süß A., Bischoff G., Mueller A., Buhr L. (2006). Chemical and biological monitoring of the load of plant protection products and of zoocoenoses in ditches of theorchard region “Altes Land”. Nachrichtenbl. D. Pflanzenschutzd.

[2] Lorenz S., Mueller A. (2018, December14). Three-year zooplankton monitoring in ditches of the apple orchard region ‘Altes Land’. Open Agrar Repos..

[3] Lorenz S., Bischoff G., Stähler M. (2018, February 13). Three-year pesticide monitoring in ditches of the apple orchard region ‘Altes Land’. Open Agrar Repository.

[4] Lorenz S., Bischoff G., Stähler M. (2018). Data on three-year pesticide monitoring in ditches of the apple orchard region of Altes Land, Germany. Data in Brief.

[5] Dodson S. (1992). Predicting crustacean zooplankton species richness. Limnol. Oceanogr..

[6] Cuker B.E. (1997). Field experiment on the influence of suspended clay and P on the plankton of a small lake. Limnol. Oceanogr..

[7] Yan N.D., Keller W., Somers K.M., Pawson T.W., Girard R.E. (1996). Recovery of crustacean zooplankton communities from acid and metal contamination: comparing manipulated and reference lakes. Can. J. Fish. Aquat. Sci..

[8] Chiba S., Batten S., Martin C.S., Ivory S., Miloslavich P., Weatherdon L.V. (2018). Zooplankton monitoring to contribute towards addressing global biodiversity conservation challenges. J. Plankton Res..

